# Taliacotian Operation

**Published:** 1852-06

**Authors:** Joseph Pancoast

**Affiliations:** Prof. of Anatomy in Jefferson Medical College


### MEDICAL EXAMINER,

AND

### RECORD OF MEDICAL SCIENCE.

NEW SERIES.—NO. XC.—JUNE, 1852.

---

### ORIGINAL COMMUNICATIONS.

---

*Taliacotian Operation.* By JOSEPH PANCOAST, M. D., Prof. of Anatomy in Jefferson Medical College. Reported by CHARLES P. TURNER, M. D., of Philadelphia.

Mr. E—— C——, of Pittsburgh, having been, during the past winter, the subject of one of the most successful rhinoplastic operations ever performed, requests that the particulars of his case be presented to the profession through the pages of the Philadelphia Medical Examiner.

The subject of the operation about to be described is a stout, plethoric man, of sanguine temperament, æt. 45. About fourteen years ago he suffered from a severe bilious attack, in the treatment of which, a violent mercurial disease was superinduced, terminating in the destruction of nearly the entire nasal structure. The small remnants of the alæ had collapsed, and united by granulation, forming an irregular ridge, scarcely prominent above the facial level; while the two nostrils, deprived of their septum, formed a single oval orifice externally. The deformity was great, and the source of extreme anoyance to the patient. He had worn, at different times, an artificial nose, ingeniously constructed by an Italian artist, but the irritation to the cuticle, and the obstruction to free respiration occasioned by it, had caused him to discontinue its use. During the past winter he applied to Prof. Pancoast, who decided to perform at once a plastic operation for the relief of the deformity. The operation was performed before the Class in the amphitheatre of the Jefferson Medical College, on the fourth of February. Preparatory to the operation, a piece of leather corresponding to the size of the nose wanted, was cut somewhat in the shape of a lateral half of a pair of bellows, the handle of the bellows corresponding to the projecting strip about to form the columna nasi. This model was then spread out on the forehead with the base uppermost, and its boundaries accurately marked out on the surface with ink. Care was taken that the model should be sufficiently large, as the complete success of this operation depends, in a manner, upon there being flap enough to allow for all subsequent contractions. The patient being then put under the influence of ether, the remains of the old nose were carefully pared, and the margins of the nasal aperture were cut into deep, narrow grooves. The flap of skin marked out on the forehead was then dissected up, together with all the areolar tissue down to the periosteum, so that the flap hung merely attached by a narrow strip between the eyebrows. Several small arteries bled freely, requiring ligation. After all bleeding had been arrested, the flap was twisted upon itself, so as to bring the raw surfaces into opposition; and the edges, after being bevelled off, were fitted into the edges previously prepared for their reception, and fastened with the plastic suture of Dr. Pancoast.*

The septum was also fitted into a transverse groove cut at right angles to the median line, and drawn firmly into its place by the suture. The nose thus formed was then carefully dressed, internally with greased lint, externally with strips of the same; together with the usual warm water top dressing. The patient was then removed to the Infirmary adjacent to the operating

*For a description of this suture, see Pancoast's Operative Surgery, 2d edition, p. 353.

room, where he was at once put to bed, and the following formula ordered:

℞. Liq. Potass. Citrat.
Liq. Ammoniæ Acet. *aa* f.℥iii.
Antim. et Potass. Tart.
Morphiæ Acetat. *aa* gr. i.
Spirit Etheris Nitrici, f℥ii.
M. S. Take a tablespoonful every hour.

Under the use of this preparation, attention being paid also to the condition of the bowels; the case progressed most favorably until Sunday the 8th, four days after the operation. On this morning the sutures and ligatures were removed by Dr. P., the parts being entirely united. The dressing was now changed. Strips of adhesive plaster were applied over graduated compresses, so as retain the nose in a proper position. Firm rolls of oiled lint were also introduced into the nostrils to prevent collapse. During the afternoon febrile symptoms set in, which were followed in the evening with traumatic delirium. The patient, imagining that some enemy was seeking his life, attempted to throw himself from the window of his room. He was restrained with difficulty by the physician in attendance, and was at last quieted by opiate injections and anodynes internally administered. Towards midnight he fell asleep and rested quietly till morning. Subsequently, the case progressed without an unfavorable symptom. The nose was carefully modelled at every dressing. Occasionally slight granulations sprung up at the alar junctions, which were speedily removed by the Argent. Nitras. The wound in the forehead was treated with the cold water dressing and gradually filled up by the modelling process of Macartney, leaving very little scar. During the seventh week of treatment, the nostrils were trimmed with a straight bistoury into a symmetrical shape; silver tubes were also made from wax models, and fitted into the nostrils. A gutta percha shield appropriately shaped, was fitted closely over the nose, and so fastened with straps so as to prevent all motion of the new member. At the commencement of the eighth week, at the time of the patient's discharge, the appearance of the nose may be thus briefly described: Anterior surface smooth and uniformly rounded; superior junction slightly irregular, the prominence caused by the twisting of the flap not having entirely disappeared; lateral edges regular, showing very little scar, except at the upper part of the right margin, where it joined the prominence caused by the twist; inferior surface firm, with the edges of the nostrils smoothly turned in; septum slightly prominent, yet firm, and capable of affording considerable support as a columna nasi. In short, the results of the operation have been most satisfactory, not only to the patient himself but to all those who carefully noticed the progress and successful termination of the case. A similar operation, equally successful, was performed last summer by Dr. P. in the case of a young lady from North Carolina. In each of the *nine* rhinoplastic operations hitherto attempted by Dr. Pancoast, the parts have uniformly united by the first intention, and success is mainly to be attributed to the superiority of the *plastic* suture over every other form of ligature.

